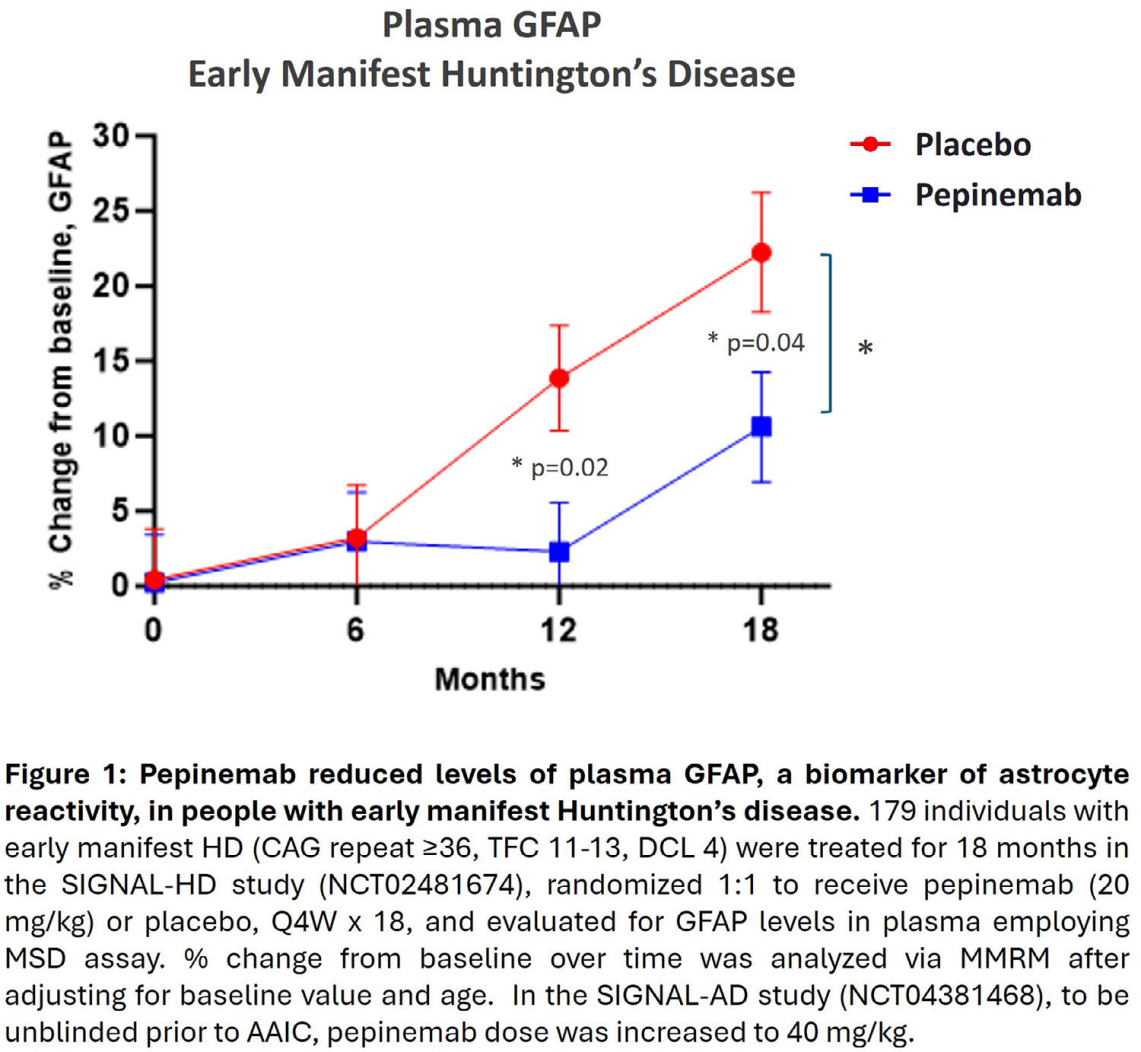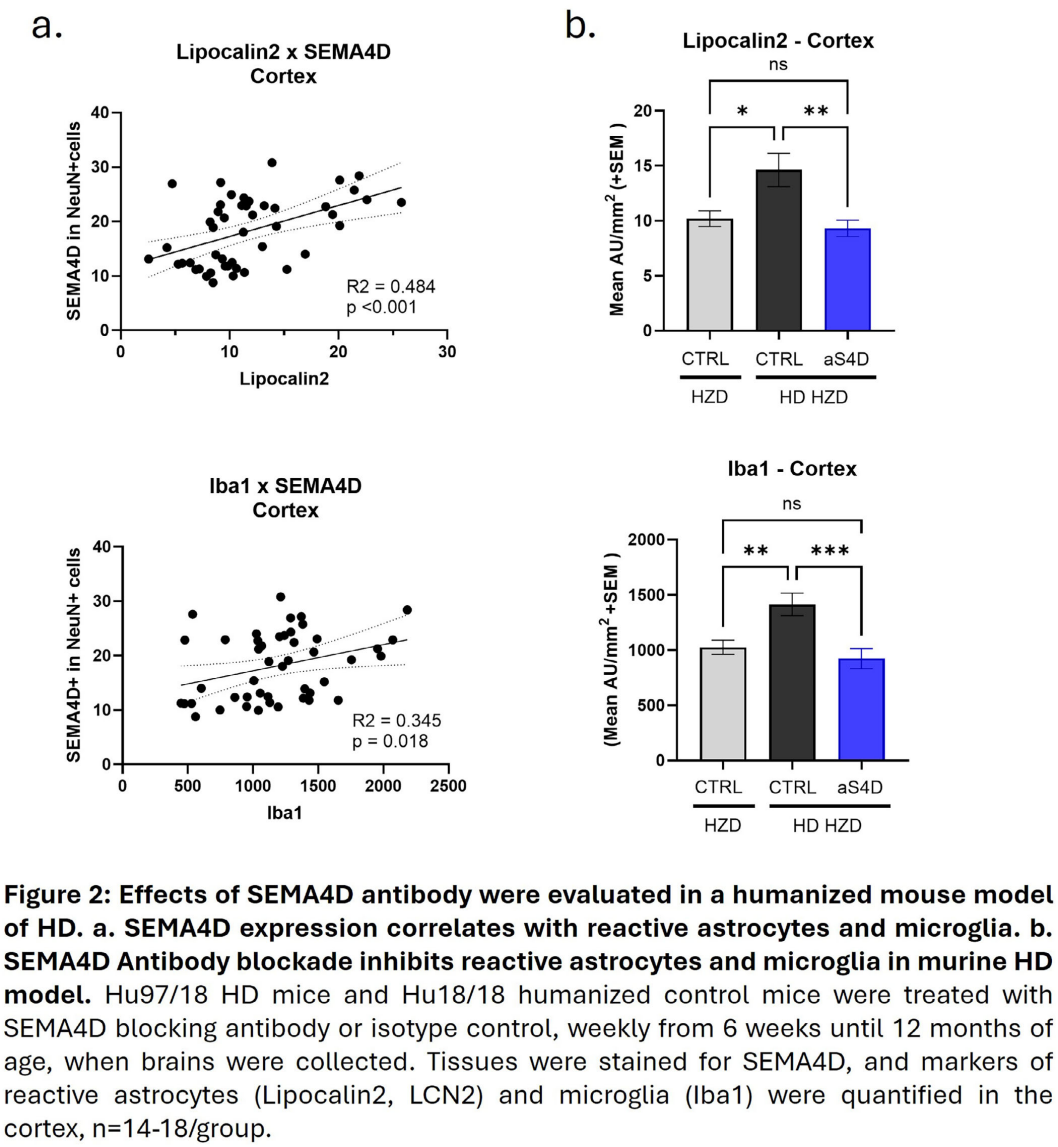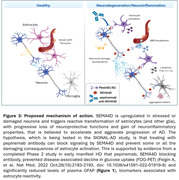# Results of SIGNAL‐AD, a randomized, phase 1b/2 trial to evaluate safety and efficacy of targeting reactive astrocytes with pepinemab, SEMA4D blocking antibody, in people with mild Alzheimer’s dementia

**DOI:** 10.1002/alz.092538

**Published:** 2025-01-09

**Authors:** Eric R Siemers, Elizabeth E Evans, Terrence L Fisher, Megan Boise, Amber Foster, John E Leonard, Vikas Mishra, Crystal L Mallow, Anton P. Porsteinsson, Raymond Scott Turner, Wendy Bond, John Huffaker, Martin R. Farlow, Lawrence S. Honig, Elizabeth Sheldon, Ryan A Townley, Paul Winner, Jeffrey Norton, Richard Holub, Paayal Patel, David S. Geldmacher, Horatio Capote, Stephen Thein, Maurice Zauderer

**Affiliations:** ^1^ Siemers Integration LLC, Zionsville, IN USA; ^2^ Vaccinex, Inc., Rochester, NY USA; ^3^ University of Rochester School of Medicine and Dentistry, Rochester, NY USA; ^4^ Georgetown University Medical Center, Washington, DC USA; ^5^ Neuropsychiatric Research Center of Southwest Florida, Fort Myers, FL USA; ^6^ Neuropsychiatric Research Center of Southwest Florida, Stuart, FL USA; ^7^ Indiana Alzheimer's Disease Research Center, Indianapolis, IN USA; ^8^ Columbia University Irving Medical Center, New York, NY USA; ^9^ JEM Research, Atlantis, FL USA; ^10^ University of Kansas Medical Center, Kansas City, KS USA; ^11^ Premiere Research Institute, West Palm Beach, FL USA; ^12^ Charter Research, Lady Lake, FL USA; ^13^ Neurological Associates of Albany, Albany, NY USA; ^14^ Brain Matters Research, Delray Beach, FL USA; ^15^ University of Alabama at Birmingham, Birmingham, AL USA; ^16^ Dent Neurologic Institute, New York, NY USA; ^17^ Pacific Research Center, Newark, CA USA

## Abstract

**Background:**

The earliest recognized biomarker of AD is deposition of Aβ amyloid that leads to formation of plaques and may, over time, trigger or at least be followed by gliosis/neuroinflammation and neurofibrillary tangles, accompanied by neurodegenerative changes including neuronal and synaptic loss. We have previously reported that semaphorin 4D (SEMA4D), the major ligand of plexin B receptors expressed on astrocytes, is upregulated in diseased neurons during progression of AD and Huntington’s disease (HD). Binding of SEMA4D to PLXNB receptors triggers astrocyte reactivity, leading to loss of neuroprotective homeostatic functions, including downregulation of glutamate and glucose transporters (doi:10.1186/s12974‐022‐02509‐8), and gain of neurotoxic processes, including release of inflammatory mediators and further activation of phagocytic glia.

Pepinemab, a high affinity, SEMA4D blocking antibody, demonstrated clinical benefit and was well tolerated in a completed Phase 2 study in early manifest HD (doi:10.1038/s41591‐022‐01919‐8). Pepinemab prevented disease‐associated metabolic decline in glucose uptake (FDG‐PET) and significantly reduced levels of plasma GFAP, biomarkers of astrocyte reactivity. Importantly, pepinemab appeared to slow cognitive decline in HD by 36% (p=0.007), as determined by the HD Cognitive Assessment Battery composite score (2).

**Methods:**

The double‐blind, placebo‐controlled SIGNAL‐AD (NCT04381468) study enrolled 50 individuals with MCI or mild AD dementia (MMSE 17‐26) and amyloid positive status. Treatment was randomized 1:1 to pepinemab (40 mg/kg) or placebo (Q4W, IV infusion) for 12 months. The primary objective of the study is safety and tolerability, and key efficacy objective is evaluation of a biomarker for brain metabolic activity, FDG‐PET imaging. Additional secondary and exploratory assessments include fluid biomarkers, plasma GFAP and pTau‐217, and several validated, clinically meaningful cognitive scales (including CDR‐SB, iADRS, ADAS‐Cog13), as well as multiplex proteomics to evaluate neuroinflammatory activity.

**Results:**

Topline data, including safety, brain metabolism (FDG‐PET), fluid biomarkers of reactive astrocytes (GFAP), tau phosphorylation (p‐tau 217), and inflammation, and potential treatment effects on cognitive decline will be presented.

**Conclusions:**

Given the many physiological parallels between glial activation and inflammatory processes in HD and AD, prior results from the SIGNAL‐HD trial suggest that preventing astrocyte activation and reducing brain inflammation with pepinemab treatment could be an attractive alternative or complement to anti‐Aβ antibodies.